# The Life and Death of *Anaplasma*

**DOI:** 10.3201/eid1712.AD1712

**Published:** 2011-12

**Authors:** Setu Vora

**Affiliations:** Author affiliation: Pulmonary and Critical Care Medicine, Norwich, Connecticut, USA

**Keywords:** Anaplasma, bacteria, humans, ticks, tickborne illness, life, death

This is the dark saga of *Anaplasma phagocytophilum*
That lurks inside white-footed mice and white-tailed deer.
Ferried by blood-thirsty ticks in the sticks,
This *Anaplasma* soul is passed on to its bodily incarnations—
Larva to nymph to tick.
A new generation of infected vampires is born.
Our love for the outdoors encroaches on tick territory.
A tick bite injects *Anaplasma* storming defender neutrophils,
Using MSP2 hooks to scale the walls.
Zombie neutrophils forget to defend—the aliens multiply into a morula.
Natural killer cells spew IFN-γ to inflame the fire
That reaches a feverish frenzy of cell death.
Now whether the man lives or dies, it is the end of the road for *Anaplasma*.
It has reached *Moksha* liberation from the cycles of death and rebirth.

